# Fecal microbiota transplantation alleviated Alzheimer’s disease-like pathogenesis in APP/PS1 transgenic mice

**DOI:** 10.1038/s41398-019-0525-3

**Published:** 2019-08-05

**Authors:** Jing Sun, Jingxuan Xu, Yi Ling, Fangyan Wang, Tianyu Gong, Changwei Yang, Shiqing Ye, Keyue Ye, Dianhui Wei, Ziqing Song, Danna Chen, Jiaming Liu

**Affiliations:** 1 Department of Neurology, The Second Affiliated Hospital and Yuying Children’s Hospital of Wenzhou Medical University, Wenzhou, Zhejiang 325027 China; 2 Department of General Surgery, The Second Affiliated Hospital and Yuying Children’s Hospital of Wenzhou Medical University, Wenzhou, Zhejiang 325027 China; 3 Department of Pathophysiology, School of Basic Medicine Science, Wenzhou Medical University, Wenzhou, Zhejiang 325035 China; 4 Department of Preventive Medicine, School of Public Health and Management, Wenzhou Medical University, Wenzhou, Zhejiang 325035 China

**Keywords:** Molecular neuroscience, Depression

## Abstract

Alzheimer’s disease (AD) is the most common dementia in the elderly. Treatment for AD is still a difficult task in clinic. AD is associated with abnormal gut microbiota. However, little is known about the role of fecal microbiota transplantation (FMT) in AD. Here, we evaluated the efficacy of FMT for the treatment of AD. We used an APPswe/PS1dE9 transgenic (Tg) mouse model. Cognitive deficits, brain deposits of amyloid-β (Aβ) and phosphorylation of tau, synaptic plasticity as well as neuroinflammation were assessed. Gut microbiota and its metabolites short-chain fatty acids (SCFAs) were analyzed by 16S rRNA sequencing and ^1^H nuclear magnetic resonance (NMR). Our results showed that FMT treatment could improve cognitive deficits and reduce the brain deposition of amyloid-β (Aβ) in APPswe/PS1dE9 transgenic (Tg) mice. These improvements were accompanied by decreased phosphorylation of tau protein and the levels of Aβ40 and Aβ42. We observed an increases in synaptic plasticity in the Tg mice, showing that postsynaptic density protein 95 (PSD-95) and synapsin I expression were increased after FMT. We also observed the decrease of COX-2 and CD11b levels in Tg mice after FMT. We also found that FMT treatment reversed the changes of gut microbiota and SCFAs. Thus, FMT may be a potential therapeutic strategy for AD.

## Introduction

Alzheimer’s disease (AD) is the most common dementia in the elderly. The accumulation of amyloid-β (Aβ) and downstream pathological events (such as tau hyperphosphorylation, neuroinflammation, and synaptic loss) eventually lead to a gradual decline in cognitive function^1,2^. Recent evidence has demonstrated that AD is associated with abnormal gut microbiota^3,4^. The abundance of *Helicobacteraceae* and *Desulfovibrionaceae* at the family level and *Odoribacter* and *Helicobacter* at the genus level significantly increased in APPswe/PS1dE9 transgenic (Tg) mice compared with wild-type (WT) mice^5^. Gut microbes played vital roles in the pathogenesis of AD^6,7^. The probiotic consumption positively affected cognitive function in AD patients via regulation of gut microbiota^8^. The combination of *Acidobacteria* and *Bifidobacterium* significantly delayed AD progression in 3xTg-AD mice^9^. Our previous studies demonstrated that probiotics exert positive effects on depression^10^, vascular dementia^11^, diabetes combined with cerebral ischemia^12^ and traumatic brain injury^13^ by regulating the microbiota. Furthermore, specific bacteria from gut microbes produce metabolites, such as short-chain fatty acids (SCFAs), which can exert beneficial effects against neuropsychiatric disorders. Specifically, the SCFA butyrate can alleviate cognition deficits^14^ and enhance BDNF expression^15^. Our previous studies also showed that butyrate could exert a protective effect against Parkinson’s disease^16^, depression^17^, and traumatic brain injury^18^ in mice. The levels of SCFAs are influenced by the composition of the microbiota^19^ and may change with the occurrence of gut microbial dysbiosis^20^. Considering that AD is associated with the abnormal gut microbiota, microbiota-targeted interventions, such as fecal microbiota transplantation (FMT), which is introduced gut microbiota into the host, might represent a potentially attractive therapeutic option against AD.

FMT from a healthy donor to a patient or diseased animal is a conventional therapeutic approach for the re-establishment of a healthy gut microbial community and has been shown to have beneficial effects^21^. Recent evidence has demonstrated that FMT from healthy donors reduced alcohol-induced anxiety and depression in an animal model of chronic alcohol exposure^22^. FMT from resilient rats to antibiotic-treated pseudo-germ-free mice significantly improved pain and depression-like phenotypes^23^. FMT reduced gut microbial dysbiosis and alleviated physical impairment in PD mice^24^. FMT have neuroprotective effects. However, the effect of FMT on AD by regulating gut microbiota remain fully unclear.

In this study, we therefore aimed to further elucidate the neuroprotective effects of FMT on AD. To address this, we performed a comprehensive exploration of cognitive impairment, Aβ accumulation, synaptic dysfunction, neuroinflammation, as well as the changes of gut microbiota and its metabolites in APPswe/PS1dE9 transgenic mice.

## Materials and methods

### Animals

Male APPswe/PSEN1dE9 transgenic (Tg) mice, aged 6 months, and nontransgenic wild-type (WT) mice were obtained from the Model Animal Research Center of Nanjing University (Nanjing, China). The mice were housed in a controlled environment at standard room temperature under a 12:12-h light/dark cycle and had access to standard food and water ad libitum. All experiments were approved and conducted in accordance with the guidelines of the Institutional Animal Care Committee of Wenzhou Medical University, China.

### Experimental design

Tg mice were randomly divided into two groups (*n* = 8 per group): (1) AD model group (Tg) and FMT treatment group (Tg + FMT). WT mice were used as the control group. The mice of Tg + FMT group were intragastrically administered with 0.2 ml fresh fecal solution of WT mice once daily for 4 weeks. For FMT experiments, 200 mg of stool (from WT mouse pellets) was daily collected from WT mice and resuspended in 5 ml of PBS mixed with sterile normal saline, and then passed through a 20 mm filter to remove large particulate. The filtrate was centrifuged at 3000 × *g* for 15 min and dissolved in physiological saline with a concentration of 400 mg/ml for transplantation, as previously described^25,26^. Before FMT treatment, mice were intragastrically administered with triple antibiotics (1.25 mg/L vancomycin, 2.5 mg/L ampicillin, and 2.5 mg/L metronidazole) in 0.2 ml/mouse daily for 3 days to remove indigenous gut microbes, as previously described with a modification^27,28^. Mice of WT and Tg were treated same dose of physiological saline.

### Behavioral tests

After the 4 weeks, Morris water maze (MWM) test and object recognition test (ORT) were performed to evaluate the cognitive function in mice.

#### MWM test

The MWM test was performed to assess spatial learning and memory ability using a cylindrical water tank (diameter 120 cm, height 50 cm, water depth 30 cm) filled with water with a video capture system (DigBehv, Jiliang, China). The MWM was hypothetically divided into four quadrants. In the third quadrant, there was a diameter 6 cm platform hidden 1.0 cm under the water surface. During 5 consecutive days of continuous training, mouse was trained to locate the hidden platform from the starting point with the deadline of 60 s. If the mouse did not find the platform within 60 s, it was allowed to stay on the platform for 10 s. On the 6th day, the platform was removed from the tank and the probe trial was performed. The mouse was allowed to swim freely for 60 s and the numbers of the platform crossing and the time spent in target quadrant was recorded.

#### ORT

ORT is based on the tendency of mice to discriminate a familiar from new object. The test was conducted 5 consecutive days and consisted of three phases: habituation, training and testing. Initially, mice were individually habituated to an open-field box (25 cm × 25 cm × 35 cm) for two consecutive days. During the training phases, on days 3 and 4, two objects of the same material were placed in a symmetric position in the center of the area. On day 5, mice were returned to the area with one familiar object (3 cm × 2 cm × 3 cm cuboid) and one novel object (2 cm in diameter and 3 cm in height cylinder). The mouse was allowed to freely explore the object for 5 min at each time. All objects and apparatus were cleaned with 70% ethanol after each trial to eliminate residual odor. The time spent exploring the familiar object (TF) and the time spent exploring the novel object (TN) were recorded and analyzed. The exploration was defined as sniffing (within 1 cm), pawing, or biting the object, but not leaning against or standing on the object. A discrimination index (DI) was calculated as follows: DI = (TN − TF/TN + TF) × 100%.

### Tissue collection

After the behavioral tests, mice were deeply anesthetized with 10% chloral hydrate (5 μl/g) and euthanized by cervical dislocation. We quickly dissected the brains and separated the cortex and hippocampus, then placed them in liquid nitrogen for rapid freezing and stored at −80 °C for western blotting. At the same time, tissue samples of the brain from mice in each group were collected and fixed, paraffin-embedded, and sectioned at 5 μm for pathological staining. Fecal samples were obtained and stored at −80 °C for 16S rRNA gene sequencing and nuclear magnetic resonance (NMR) spectroscopy.

### Analysis of Aβ40 and Aβ42 levels by ELISA

The hippocampus samples of brain were homogenized and used to measure the concentration of Aβ40 and Aβ42 by using ELISA kit (Invitrogen Corporation, Camarillo, California, USA) according to the instructions. The standard curve was established and then used to calculate the levels of Aβ40 or Aβ42 in the tissues. The obtained values were corrected for the wet weight of brain sample and expressed as μg/mg.

### Western blotting

The cortex samples of brain were homogenized using a handheld homogenizer in RIPA lysis buffer consisting of 50 mM Tris (pH 7.4), 150 mM NaCl, 1% sodium deoxycholate, 0.1% SDS, and multiple inhibitors. The homogenate was incubated on ice for 20 min and centrifuged at 12,000 × *g* for 20 min at 4 °C. An enhanced bicinchoninic acid (BCA) protein assay kit was used to determine the protein concentration. The samples were separated using 12% sodium dodecyl sulfate-polyacrylamide gel electrophoresis (SDS-PAGE) and electro-transferred onto a PVDF membrane. The membrane was blocked in 5% non-fat milk for 2 h at room temperature and then incubated with phospho-Tau231 (1:1000, Bioworld, Minnesota, USA), PSD-95 (1:1000, Bioworld, Minnesota, USA), and synapsin I (1:1000, Bioworld, Minnesota, USA) and COX-2 (1:1000, Bioworld, Minnesota, USA) at 4 °C overnight. Subsequently, the membrane was washed in PBST and incubated with the appropriate HRP-conjugated secondary antibody (1:2000, Beyotime, China) for 1 h at room temperature. Images were scanned and the results were quantified. β-Actin and GAPDH was used as the loading control.

### Pathological staining

Brain tissues were quickly dissected and fixed in 4% paraformaldehyde for 72 h after perfusion with 0.1 mol/L phosphate buffer (containing 4% paraformaldehyde), then dehydrated with ascending grades of ethyl alcohol, and cleaned in xylene and embedded in paraffin. The paraffin-embedded tissues were sliced to a thickness of 5 μm and dried at 65 °C. Slices were stained with Congo red staining solution (1:100, Solarbio, Beijing, China) for 10 min, drip differentiation solution for about 20 s, controlled by microscope, washed 5 min; hematoxylin light dyeing for about 10 s. The gradient alcohol was dehydrated, the xylene was transparent, and the neutral gum was sealed. Immunohistochemical staining was performed. PSD-95 (1:400, Bioworld, Minnesota, USA), synapsin I (1:200, Bioworld, Minnesota, USA), COX-2 (1:200, Bioworld, Minnesota, USA), and cluster of differentiation 11b (CD11b, 1:200, Southern Biotech, Birmingham, AL 35209, USA) were applied overnight at 4 °C and incubated with HRP-conjugated secondary antibodies and the sections were visualized using DAB as the chromogen.

### Fecal DNA extraction and 16S rRNA gene sequencing

After all behavioral tests, animals were anesthetized. Fresh colonic fecal samples (0.2–0.3 g per sample) were collected from the mice and immediately stored at −80 °C. DNA extraction was performed using a QIAGEN stool DNA extraction kit (QIAGEN, Hilden, Germany) according to the manufacturer’s instructions. For analysis of the phylogenetic composition of the gut microbiota, the V3–V4 region of the 16S rRNA gene was amplified using the Illumina MiSeq 2500 platform (Shanghai Majorbio Biopharm Technology Co., Ltd). A beta diversity distance matrix was computed from the previously constructed OTU table using UniFrac analysis. Principal component analysis (PCA), principal coordinate analysis (PCoA), a heatmap of RDA-identified key OTUs, and the unweighted pair group method with arithmetic mean (UPGMA) to analyze the beta diversity. The 16S rRNA sequencing data set generated by the MiSeq platform was merged and decomposed into separate samples using QIIME version 1.9.0. OTUs were selected, and USEARCH V7 was used to search the Greengenes Database version 13.8, with a sequence similarity of 97%. OTUs accounting for <0.005% of the total number of sequences were excluded. The characteristics of the gut microbiota were analyzed by linear discriminant analysis (LDA) effect size (LEfSe) for biomarker discovery. LEfSe detects the features with significant differences in abundance using the Kruskal–Wallis rank sum test and applies LDA to evaluate the effect size of each feature.

### SCFA analysis by nuclear magnetic resonance (NMR) spectroscopy

Fresh colonic fecal samples (50–60 mg per sample) were collected from mice and immediately extracted using 1 ml phosphate buffer (pH 7.4) with 145.1 μM TSP-d4 for chemical shift referencing (δ 0.00). The samples were frozen and thawed three times with liquid nitrogen, then homogenized (6500 rpm, 1 cycle, 60 s) and centrifuged (11,000 × *g*, 4 °C, and 10 min). We transferred the supernatant to a new test tube and then added 600 μL PBS to the pellets. The fecal supernatant was separated by centrifugation (11,000 × *g*, 4 °C, and 10 min). A total of 600 μl of spiked fecal extract was transferred to a 5 mm NMR tube (Norell, Morganton, NC). ^1^H NMR spectra were obtained at 600.13 MHz on a Bruker AVANCE III 600 MHz NMR spectrometer with a 5-mm TXI probe (Bruker BioSpin, Rheinstetten, Germany). The ^1^H NMR spectra were recorded by the standard single-pulse experiment with water signal presaturation. The main acquisition parameters were as follows: data points, 64 K; relaxation delay, 2 s; spectral width, 12,000 Hz; and acquisition time, 2.65 s per scan, as previously described^29^.

### Statistical analysis

Data are presented as the mean ± SEM. Statistical analyses were performed using SPSS statistical software, version 18.0 (SPSS, Chicago, IL, USA). The data were analyzed by two-way ANOVA or one-way ANOVA by Tukey’s test. *P* < 0.05 was considered statistically significant.

## Results

### Effect of FMT on cognitive deficits in Tg mice

**Fig. 1:**
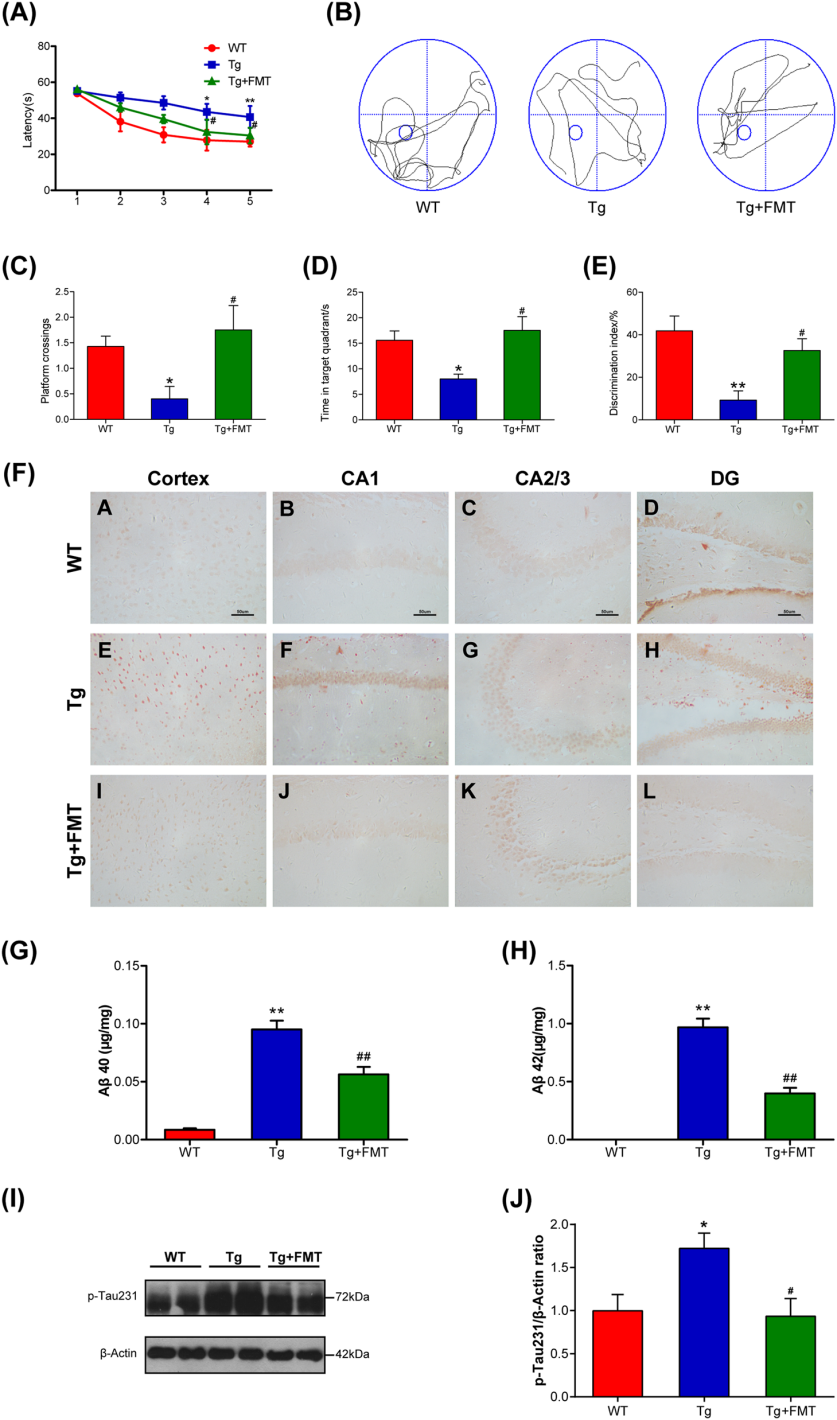
Effects of FMT on behavioral function and amyloid plaque in Tg mice. **a** Escape latency of mice for 5 consecutive days in Morris water maze test. **b** Representative swimming tracks of mice in Morris water maze test. **c** Frequency of crossing the platform of mice in Morris water maze test. **d** Detention time in target quadrant in Morris water maze test. **e** Discrimination index of the object recognition test. Error bars indicate SEM; *n* = 6–8 for each group. *P < 0.05 vs. WT group, **P < 0.01 vs. WT group, ^#^*P* < 0.05 vs. Tg group. **f** Representative photographs of Congo red staining in the cortex and hippocampal regions (CA1, CA3, and DG). CA, cornu ammonis; DG, dentate gyrus; magnification ×400. Scale bar = 50 µm. **g** The level of Aβ_40_ in the brain. **h** The level of Aβ_42_ in the brain. **i** Western blot analysis of p-Tau expression. **j** Quantitative analysis ratio of p-Tau/β-actin, the reference value of the p-Tau/β-actin was the ratio of the WT group. Error bars indicate SEM; *n* = 4–6 for each group. *P < 0.05 vs. WT group, **P < 0.01 vs. WT group, ^#^*P* < 0.05 vs. Tg group, ^##^*P* < 0.01 vs. Tg group

The cognitive function of mice was assessed by the MWM test and the ORT. The MWM test is conventionally used to measure cognitive function. In the training trials, all groups gradually learned to seek the hidden platform and gradually shortened their escape latency to reach the platform (Fig. 1a). The Tg mice exhibited prolonged escape latency compared with the WT mice (4th: *P* < 0.05, 5th: *P* < 0.01, Fig. 1a). Mice in the Tg + FMT group displayed shorter escape latencies than the Tg group on the 4th day and the 5th day (4th: *P* < 0.05, 5th: *P* < 0.05, Fig. 1a). Representative swimming paths of mice are illustrated in Fig. 1b. The Tg mice stayed in the target quadrant for a short time and crossed through it fewer frequency than the WT mice (*P* < 0.05, Fig. 1c, d). The frequency and time spent in the target quadrant were much greater in the FMT treatment group than in the Tg group (*P* < 0.05, Fig. 1c, d), indicating that FMT treatment was able to attenuate the impairment of spatial learning ability in Tg mice. In the ORT, the mice in the FMT treatment group also performed better than the Tg group (*P* < 0.05, Fig. 1e), as reflected by discrimination between the familiar and novel objects and a higher discrimination index in the FMT-treated mice.

### Effect of FMT on amyloid plaque and p-Tau level in Tg mice

To investigate whether FMT affected Aβ deposition in the Tg mice, we performed Congo red staining for compact amyloid plaques^30^. The number of brick-red patches distributed in the cortex and the hippocampus (CA1, CA2/3, and DG) was counted in Congo red-stained brain tissue. The number of brick-red patches in the cortex and hippocampal regions (CA1, CA2/3, and DG) was higher in the Tg group than in the WT group (Fig. 1f). However, the number of brick-red patches was lower in the Tg + FMT group than in the Tg group (Fig. 1f), suggesting that FMT could result in a reduced amyloid plaque burden. Aβ is the main component of amyloid plaques. The Aβ40 level in the Tg group was significantly higher than the level in the WT group (*P* < 0.01, Fig. 1g), whereas the level of Aβ40 in FMT-treated mice was dramatically decreased compared with the level in the Tg mice (*P* < 0.01, Fig. 1g). Similarly, the level of Aβ42 in FMT-treated mice was dramatically decreased compared with the level in the Tg mice (*P* < 0.01, Fig. 1h). These results suggested that the amyloid burden could be altered in the Tg mice following treatment with FMT, based on the reduction in the Aβ42 and Aβ40 levels in the Tg mice. Western blots showed that Tau phosphorylation at the threonine 231 site was significantly decreased in the FMT-treated mice (Fig. 1i, j). These findings indicated that FMT could suppress Aβ accumulation and Tau hyperphosphorylation in Tg mice.

### Effect of FMT on synaptic plasticity in Tg mice

**Fig. 2:**
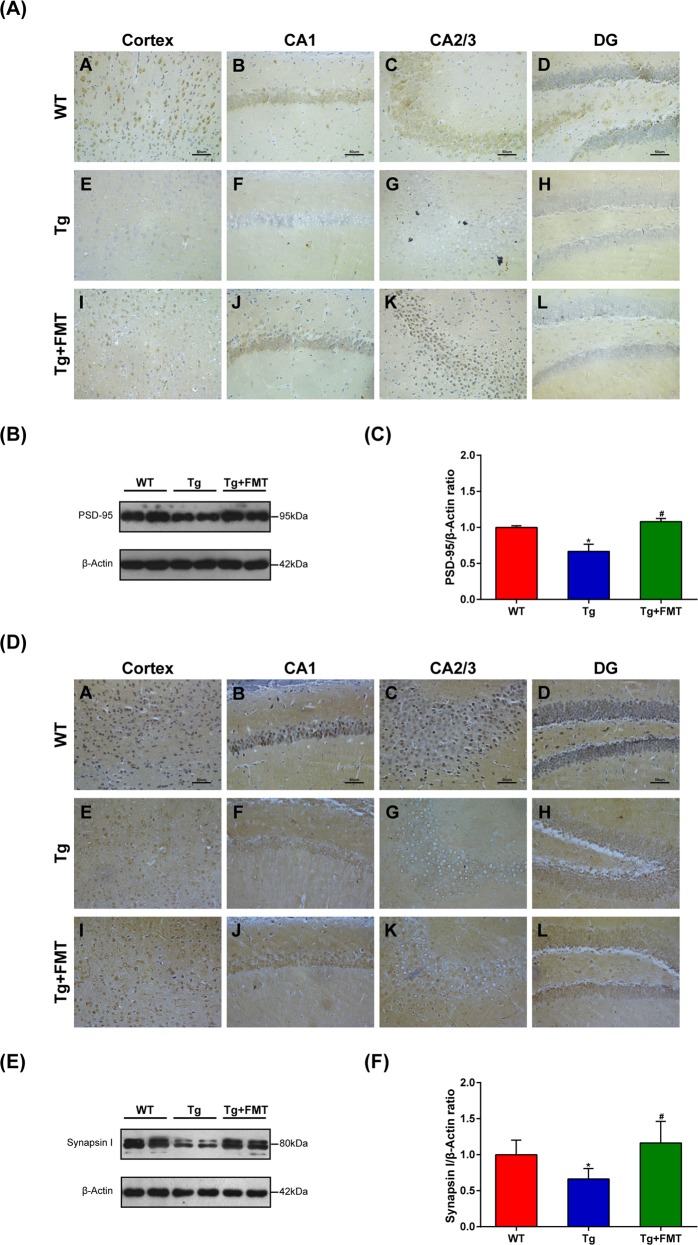
Effects of FMT on levels of PSD-95 and synapsin I in Tg mice. **a** Immunohistochemical analysis of PSD-95 in the cortex and hippocampal regions (CA1, CA3, and DG); Magnification × 400. Scale bar = 50 µm. **b** Western blot analysis of PSD-95 expression; (**c**) quantitative analysis of expression of PSD-95, the reference value of the PSD-95/β-actin was the ratio of the WT group. **d** Immunohistochemical analysis of synapsin I in the cortex and hippocampal regions (CA1, CA3, and DG); Magnification × 400. Scale bar = 50 µm. **e** Western blot analysis of synapsin I expression; (**f**) quantitative analysis of expression of synapsin I, the reference value of the synapsin I/β-actin was the ratio of the WT group. Error bars indicate SEM; *n* = 4–6 for each group. *P < 0.05 vs. WT, ^#^*P* < 0.05 vs. Tg

To examine whether FMT affected synaptic function in the Tg mice, we assessed synaptic plasticity-related proteins, including PSD-95 and synapsin I, by immunohistochemistry and Western blot. In immunohistochemistry, the level of PSD-95 in the cortex and hippocampal regions of the Tg group were lower than those in the WT group. However, the level of PSD-95 in the FMT group was higher than in the Tg group (Fig. 2a). Western blots further showed that the level of PSD-95 was significantly decreased in the Tg group compared with the WT group (*P* < 0.05, Fig. 2b, c) and significantly increased in FMT-treated mice (*P* < 0.05, Fig. 2b, c). In the Immunohistochemistry, the level of synapsin I in the cortex and hippocampal regions were lower in the Tg group than in the WT group. However, the level of synapsin I in the Tg + FMT group was higher than the level in the Tg group (Fig. 2d). Furthermore, western blots showed that the level of synapsin I in the Tg group were significantly lower than those in the WT group (*P* < 0.05, Fig. 2e, f), whereas the FMT-treated mice had significantly increased level of synapsin I (*P* < 0.05, Fig. 2e, f). These findings indicated that FMT could attenuate synaptic dysfunction in Tg mice.

### Effect of FMT on COX-2 and CD11b in Tg mice

**Fig. 3:**
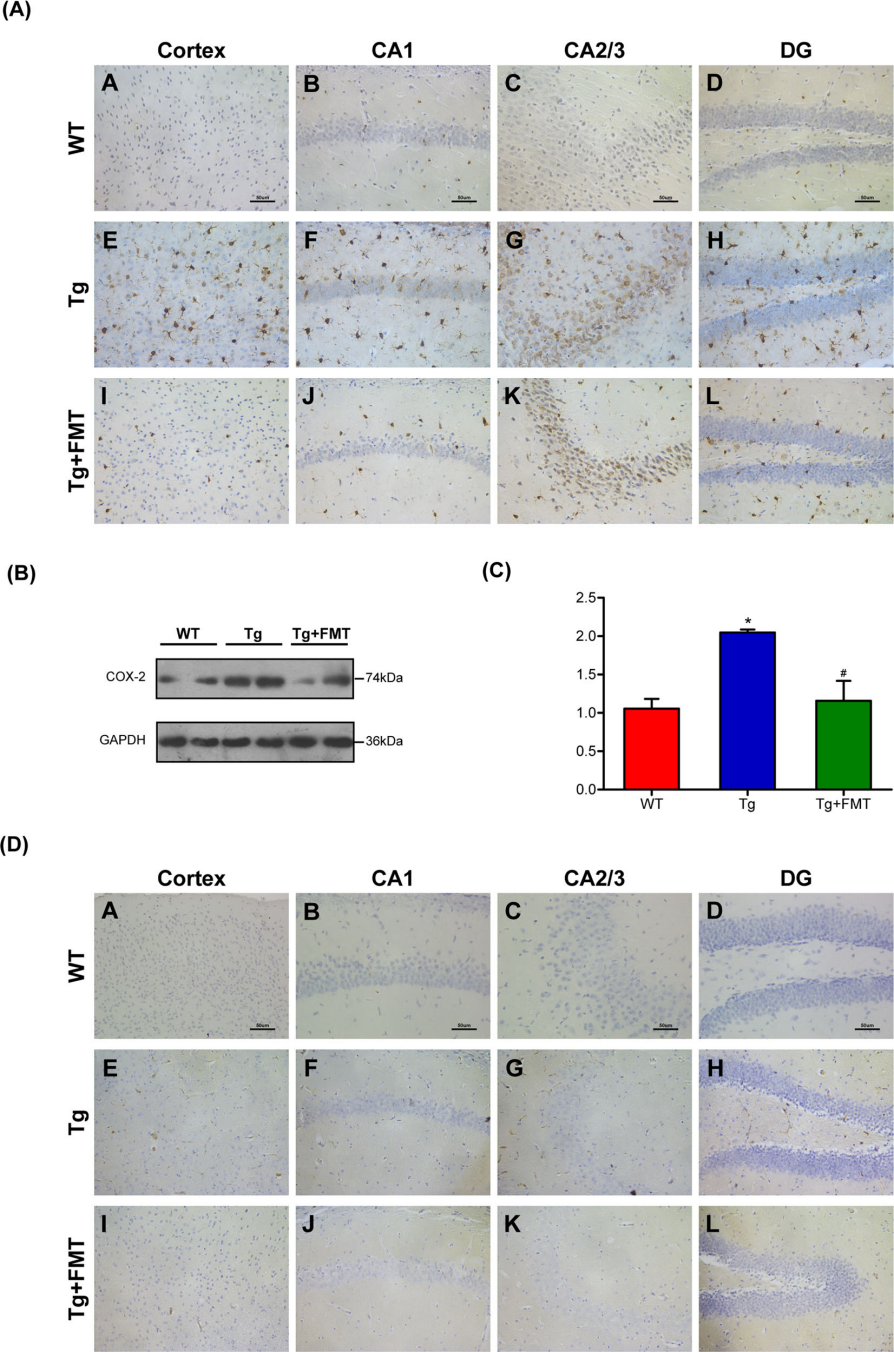
Effects of FMT on levels of COX-2 and CD11b in Tg mice. **a** Immunohistochemical analysis of COX-2 in the cortex and hippocampal regions (CA1, CA3, and DG); Magnification × 400. Scale bar = 50 µm. **b** Western blot analysis of COX-2 expression; **c** quantitative analysis of expression of COX-2, the reference value of the COX-2/GAPDH was the ratio of the WT group. Error bars indicate SEM; *n* = 4–6 for each group. *P < 0.05 vs. WT, ^#^*P* < 0.05 vs. Tg. **b** Immunohistochemical analysis of CD11b in the cortex and hippocampal regions (CA1, CA3, and DG); Magnification × 400. Scale bar = 50 µm

COX-2 protein is critical in the pathophysiological process of inflammation. The level of COX-2 in the cortex and the hippocampal regions of the Tg group were higher than those of the WT group. However, the level of COX-2 in the Tg + FMT group was lower than that in the Tg group (Fig. 3a). Western blots further showed that the level of COX-2 in the Tg group were significantly increased compared with the WT group (*P* < 0.05, Fig. 3b, c), whereas that of the FMT-treated mice were significantly decreased in the level of COX-2 (*P* < 0.05, Fig. 3b, c). The level of CD11b in the Tg group were higher than those in the WT group (Fig. 3d). However, the level of CD11b in the Tg + FMT group was lower than that in the Tg group (Fig. 3d). These findings indicated that FMT could attenuate neuroinflammation in Tg mice.

### Effect of FMT on the composition of the gut microbiota in Tg mice

**Fig. 4:**
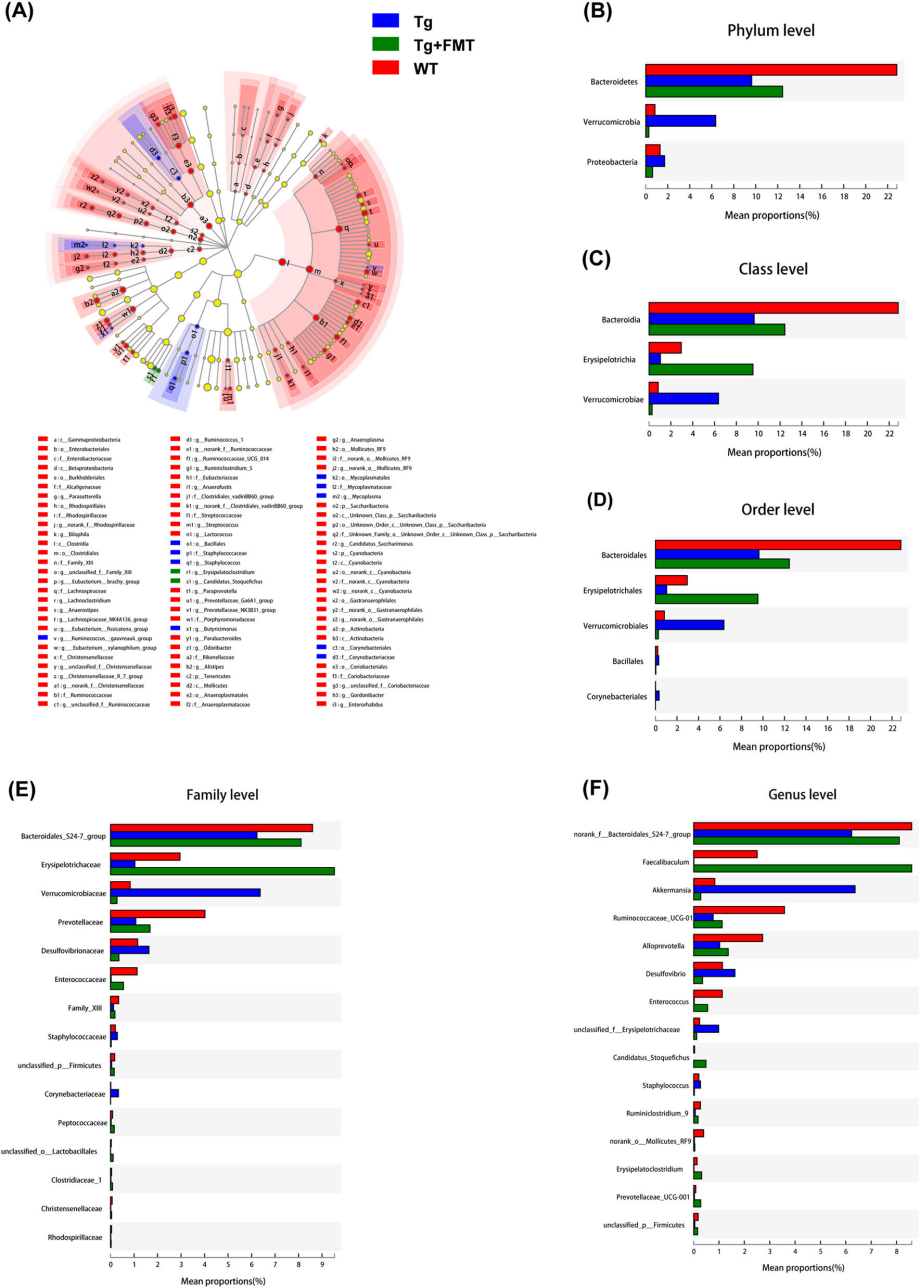
Effects of FMT on different bacterial taxa. Histogram of the LDA scores for different abundant genera (**a**), on the phylum level (**b**), the class level (**c**), the order level (**d**), the family level (**e**), the genus level-selected major taxa (**f**)

To investigate whether FMT affected the gut microbiota of the Tg mice, we performed microbiota analysis. Discriminant analyses using LEfSe showed that the treated and untreated mice had significantly different bacterial phylotypes (Fig. 4a). Analysis revealed differences in the abundance of taxa. Gut microbiota of Tg mice was characterized by an overabundance of *Proteobacteria* and *Verrucomicrobia* at the phylum level and *Akkermansia*, and *Desulfovibrio* at the genus level, as well as a low abundance of *Bacteroidetes* at the phylum level and the *Bacteroidales*_S24-7 group and *Alloprevotella* at the genus level, as displayed in Fig. 4b–f. At the phylum level, *Bacteroidetes* were enriched in the WT mice, while *Proteobacteria* and *Verrucomicrobia* were enriched in the Tg mice. The abundance of *Proteobacteria* and *Verrucomicrobia* was decreased, while that of *Bacteroidetes* was increased, in the FMT-treated mice (Fig. 4b). At the genus level, the abundance of the *Bacteroidales*_S24-7 group, *Ruminococcaceae*_UCG-01, and *Alloprevotella* were enriched in the WT mice, while *Akkermansia* and *Desulfovibrio* were enriched in the Tg mice. FMT reversed the alterations in the composition of the microbiota in Tg mice.

### NMR-based metabolic profile

**Fig. 5:**
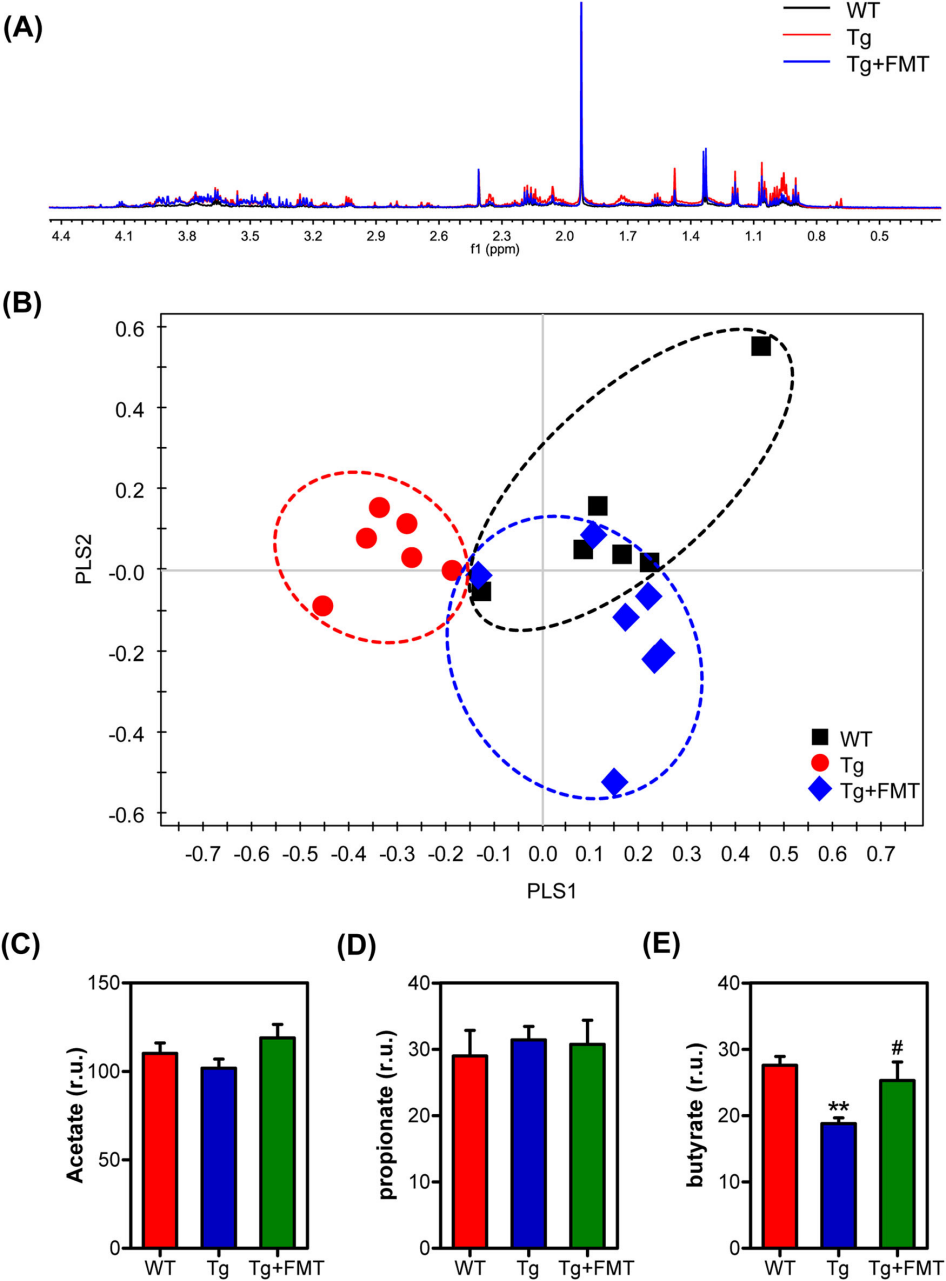
Effects of FMT on metabolic differences in the fecal samples. A representative 600 MHz ^1^H NMR spectrum of the fecal samples (**a**), and PLS-DA classification based on the normalized spectra (**b**), changes in metabolite levels: acetate (**c**), propionate (**d**), butyrate (**e**). Error bars indicate SEM; *n* = 6–8 for each group. **P < 0.01 vs. WT, ^#^*P* < 0.05 vs. Tg

The NMR spectra were normalized into corresponding tissue weights to assess the relative content of the extracted and bound metabolites to reduce the dimensionality of the data for further metabolic analysis, as reflected in the typical ^1^H NMR spectra of fecal samples and a total of three identified metabolites, the SCFAs acetate, propionate and butyrate, as shown in Fig. 5a. It was demonstrated from the PLS-DA score plot that the Tg group was separated from the other two groups along PLS1 (Fig. 5b). To examine whether FMT affected SCFAs levels in the Tg mice, we assessed the levels of acetate, propionate and butyrate. The level of butyrate was significantly increased in the FMT-treated mice (Fig. 5e), and the levels of acetate and propionate were not significantly different among the three groups (Fig. 5c, d).

## Discussion

FMT has been shown to exert beneficial effects against neuropsychiatric disorders by modulating gut microbiota, however, its effect on AD remains unclear. This study demonstrated the neuroprotective effects of FMT against AD in APPswe/PS1dE9 transgenic mice. Our results showed that FMT treatment significantly relieved cognitive deficits, Aβ accumulation, synaptic dysfunction, and neuroinflammation. These protective effects of FMT may be related to reversing alterations of gut microbiota and its metabolites SCFAs.

Cognitive deficits are the main manifestation of AD^31,32^. Improving cognitive function is a desirable target for therapies against AD. Improving cognitive deficits in AD patients^33^ and Tg mice^9^ via regulating gut microbiota is an area of active research. In this study, the data showed that FMT effectively alleviated cognitive deficits in the Tg mice as shown by the MWM test and the ORT, suggesting that FMT might be involved in alleviating cognitive deficits. The development of AD was accompanied by pathological changes. Aβ deposition is a hallmark of AD pathogenesis. Congo red staining is a reliable indicator of Aβ deposition, which is an important cause of cognitive decline in AD. Probiotics reduced the accumulation of Aβ and ameliorated the effect of Aβ toxicity^34^. In this study, the data showed that FMT treatment could decrease amyloid deposition and the levels of Aβ40 and Aβ42 in the Tg group. C-Jun N-terminal kinase (JNK) is related to the production of Aβ^35,36^. Inactivation of JNK could decrease Aβ production and the resulting cognitive deficits in AD mice^36,37^. Our results showed that FMT treatment could inhibit JNK activity in Tg mice. Taken together, our results suggested that FMT treatment could ameliorate cognitive deficits, Aβ deposition and Tau phosphorylation in Tg mice.

AD is mainly characterized by abnormal synaptic plasticity^7,38,39^, which is strongly correlated with the cognitive decline observed in AD patients^40^. The accumulation of plaques could affect synaptic activity^41,42^, which was an early pathogenic event in AD. PSD-95 and synapsin I were indicators of synaptic plasticity^43^. PSD-95 was a common marker of postsynaptic components and indirectly reflects changes in synaptic plasticity^44^. Synapsin I was a presynaptic protein that responds to neuronal activity and regulates the availability of synaptic vesicles to participate in neurotransmitter release^45^. The levels of synapsin I and PSD-95 were significantly decreased in AD mice^7,46^. In this study, FMT treatment reversed the decreased levels of PSD-95 and synapsin I in the Tg mice, indicating that FMT treatment could prevent the abnormal synaptic plasticity of AD. Neuroinflammation was thought to be an important aspect of AD pathogenesis^47^. COX-2 activated proinflammatory signaling pathways^48^, and interactions between Aβ and microglia induced the release of proinflammatory factors. FMT provided neuroprotection by inhibiting inflammation in an animal model of PD^24^. In this study, FMT treatment decreased the levels of COX-2 and CD11b in the Tg mice, indicating that FMT treatment could inhibit neuroinflammation in AD.

Alterations of gut microbiome were related to AD^3^. Gut microbiome could influence brain function via the gut-brain axis^3,4,49,50^. Recent evidence has demonstrated that gut microbiota of AD patients and animal models were altered^4,51,52^. Germ-free (GF) mice showed significantly increased brain Aβ levels after transplantation of fecal samples from AD mice^3^. Manipulating gut microbiota can influence Aβ deposition and neuroplasticity processes^53,54^. In this study, *Proteobacteria*, *Verrucomicrobia, Akkermansia*, and *Desulfovibrio* were enriched, and Bacteroidetes and *Alloprevotella* decreased in the Tg mice, whereas FMT reversed the alterations in the microbial composition. The abundance of *Proteobacteria* increases in the gut microbiome during aging^55^. AD pathogenesis in the Tg mouse model could shift the gut microbiota toward a higher abundance of *Proteobacteria*^56^. Moreover, *Proteobacteria* was related to inflammation, which was linked to AD pathogenesis^57^ and an increased risk of dementia^58^. In this study, FMT reversed the increase in *Proteobacteria* in the Tg mice. At the family level, the abundance of *Desulfovibrionaceae* was also elevated in Tg mice^56^. In addition, the family *Desulfovibrionaceae* has a significantly negative correlation with spatial learning and memory ability, active avoidance response, and object recognition memory capability^59^. Unpredictable restraint stress potentiates rotenone-induced effects in the colon, including an increase in *Akkermansia*^60^. However, the changes associated AD are not consistent across AD studies. The phylum *Verrucomicrobia*, the genus *Akkermansia* genus and certain species are increased in PD patients^61,62^. The APP/PS1 mouse model showed a distinct microbial signature, with a decreased abundance of *Akkermansia*^63^. In this study, FMT reversed the decrease in *Bacteroidetes* in the Tg mice. *Bacteroidetes* has a well-known and frequently discussed connection to AD. AD patients showed a characteristic increase in *Bacteroidetes*^64^. The abundance of *Bacteroidetes* was decreased in AD mice^65^. Another study found, in contrast to our results, that the abundance of *Bacteroidetes* increased in the microbiome of AD participants^64^. In this study, the overabundance of *Bacteroidetes* was restored in the FMT-treated Tg mice, and *Bacteroidetes* was able to trigger the generation of SCFAs^66^. In this study, the overabundance of *Proteobacteria* and *Desulfovibrio* and the low abundance of *Bacteroidetes* and *Alloprevotella* might have participated in the process of AD. Further studies are needed to identify functional metabolites.

SCFAs are among the vital chemical mediators of the gut-brain axis. SCFAs such as acetate, propionate and butyrate may exert neuroprotective effects, which are influenced by gut microbiome^67^. SCFAs may also modulate the maturation and function of microglia in the brain^68^, suggesting the potential benefits of bacteria-derived SCFAs in modulating neuroinflammatory processes associated with neurodegenerative disorders. The SCFA butyrate could exert neuroprotective effects^69^. Butyrate and propionate also interfere with Aβ_1-40_ oligomerization^70^. Bacterial generation of butyrate, as an inhibitor of histone deacetylase, was known to exert multiple effects against a variety of neuropsychiatric disorders^71^. Our previous study also demonstrated that butyrate had neuroprotective effects on depression, PD and traumatic brain injury in mice^16–18,72^. In this study, FMT treatment significantly reversed the decrease of butyrate in the Tg mice, suggesting the mechanism of anti-AD may be related to the increase of SCFAs butyrate. A further study to investigate this potential mechanism is necessary.

In summary, FMT prevented AD-like pathology in a mouse model, and these protective effects were related to reversing changes of gut microbiota and SCFAs, suggesting that FMT therapy might be a potential therapeutic strategy for AD.
